# Inequities to the access to and use of telemedicine among cancer patients in Europe: a scoping review

**DOI:** 10.3389/fpubh.2025.1483706

**Published:** 2025-05-22

**Authors:** Victoria Leclercq, Léopold Vandervliet, Tugce Schmitt, Marc Van den Bulcke, Marie Delnord

**Affiliations:** Cancer Centre, Department of Epidemiology and Public Health, Sciensano, Brussels, Belgium

**Keywords:** cancer, telemedicine, inequities and inequalities in health, Europe, digital

## Abstract

**Introduction:**

Over the last few years, telemedicine (TM) services have increasingly been used in European health and care systems. TM services include remote assistance with teleconsultation and telemonitoring. Several sources highlight that TM services may widen existing inequities in health. Therefore, this study aimed to identify barriers and facilitators contributing to inequities to TM among cancer patients in Europe.

**Methods:**

Medline (via Ovid) and Scopus databases were searched for all publications providing evidence on factors influencing the access to and use of TMs among cancer patients aged 18 and over in Europe published between January 2018 and March 2023. The PROGRESS-plus framework was used to map health equity factors in TM services among cancer patients.

**Results:**

A total of 2072 peer reviewed publications were identified and after screening, 26 articles were retained in our scoping review. Only studies focused on TM used by cancer patients through mobile or web-based applications were included. In terms of access to TM, people with lower socioeconomic status, including difficulties with having an internet connection and not having their own mobile device, and language barrier seem to have less access to TM services. For the use of TM services, a lower level of education, few digital skills and (e-)health literacy, lack of social support, age and presence of comorbidities are important determinants.

**Discussion:**

Better integration of patient needs in TM is necessary to enhance equity and allow a better implementation of TMs in European health and care systems aligned with different initiatives such European Beating Cancer Plan.

## 1 Introduction

Cancer is a major public health concern and the second leading cause of mortality across European countries after cardiovascular disease. In Europe, disparities in preventive policies and access to advanced diagnostics, treatments, and care lead to significant differences in the timeliness of cancer diagnoses and survival rates (1). The Europe’s Beating Cancer Plan (EBCP) is the European (EU) response to fight cancer and aims to tackle the entire disease pathway (2). Alongside the EBCP, the EU Commission is also working on the digital transformation of health and care, to improve access to and quality of care (3). As demonstrated during the COVID-19 pandemic, the expanded use of telemedicine (TM) services presents a new opportunity to address public health crises. However, there is still significant potential to improve digital interventions and access to care using TMs to enhance health outcomes for cancer patients across Europe (4, 5). Moreover, TMs can offer the potential to tackle already existing inequalities among cancer patients (6). This is in line with one of the aims of the Joint Action (JA) called ‘Strengthening eHealth including TM and remote monitoring for healthcare systems for CANcer prevention and care (eCAN)’ aiming to reduce cancer care inequities across 16 European countries while exploring the role of teleconsultation and telemonitoring among cancer patients.

The World Health Organization (WHO) defines TMs as “the delivery of health care services, where distance is a critical factor, by all health care professionals using information and communication technologies for the exchange of valid information for diagnosis, treatment and prevention of disease and injuries, research and evaluation, and for the continuing education of health care providers, all in the interests of advancing the health of individuals and their communities (7).” TMs include remote assistance referring to teleconsultation (i.e., remote follow-up, diagnosis or treatment of patients) and telemonitoring services recording parameters of patients (7). TMs can improve cancer prevention and care by facilitating the delivery of more efficient and effective patient-centered care (7, 8). The expected benefits of TMs are easier access to information and personalized care, more control and empowerment over their own health (7, 8).

Several sources highlighted some difficulties in the access to and use of TMs, which can widen already existing inequities in health (9–12). However, even if progress in terms of internet access and use in recent years has been substantial in Europe, there are wide disparities (13, 14). According to the latest Eurostat estimates, in 2023 93% (variation from 80 to 99%) of European households have access to the internet, including 90.2% with broadband internet access at home (13, 14). Moreover, the difference in terms of internet access within a country, i.e., between cities and rural areas, was higher in some countries such as Bulgaria, Croatia, Greece and Portugal. Then, over 93% of Europeans aged between 16 and 74 report using the internet, mainly using mobile devices (90%), followed by laptops and tablets (63%) and desktop computers (31%).

In the cancer field, as the incidence of cancer and its associated burden increases, gaps in access to cancer services are also at risk of widening. The OECD has highlighted large disparities in cancer incidence, mortality and survival (overall and by cancer subtype), especially in vulnerable groups population (1). The main factors driving these disparities are linked to: gender, socio-economic status, level of education, or having unhealthy behaviors (e.g., smokers, alcohol, obesity…), and geographical situation (place of living within a country and between countries) (1). The WHO identified, in the general population, better use and access to digital tools among people living in urban areas, of white ethnic origin and English speakers, higher education and younger (15).

Equity has been defined by the WHO as “the absence of unfair, avoidable or remediable differences among groups of people, whether those groups are defined socially, economically, demographically, or geographically or by other dimensions of inequality (e.g., sex, gender, ethnicity, disability, or sexual orientation). Health equity is achieved when everyone can attain their full potential for health and well-being” (15). A better understanding of the factors contributing to health inequities among cancer patients using TMs in Europe is needed to ensure that the application of TM among these populations does not widen already existing inequities (10, 16, 17).

One of the aims of the eCAN JA is to reduce cancer care inequities while exploring the role of TM among cancer patients Therefore, a scoping review on inequities in the access to and use of TM among cancer patients in Europe was carried out. More specifically, this study aims to identify the barriers and facilitators contributing to the access and use of TMs among cancer patients. Where “access” refers to the ability to access the resources required for digital health (i.e., an internet connection and/or having digital devices) and “use” refers to variations in the ability of different groups that have access to resources to use digital health technology (15).

## 2 Materials and methods

A scoping review allows a systematic approach to map evidence on a given topic by identifying key concepts, theories and sources; and highlighting existing gaps in research (18). Using this study design enabled us to map the barriers and facilitators to the access to and use of TM among cancer patients in Europe through thematic analyses.

### 2.1 Protocol and registration

This scoping review was conducted and reported in accordance with the Preferred Reporting Items for Systematic Review and Meta-analysis (PRISMA2020) extension for scoping reviews (18). The completed PRISMA-ScR checklist is available in Appendix 1. A protocol has been developed and published in Open Science Framework^1^. Covidence software was used to manage search results, including removing duplicates, abstract and title screening, and full-text screening. Data extraction and analyses were performed using Microsoft Excel.

### 2.2 Literature search

Scientific studies published between January 2018 and March 2023, in English reporting on factors contributing to health inequities in the use and access to TMs among cancer patients in Europe were identified via search literature in Medline (via Ovid) and Scopus. A combination of terms of Medical Subject Headings (MeSH) and keywords, including eHealth, cancer and equity, was used in the search strategy. The completed search strategies are available in Appendix 2. Additionally, a manual search within the bibliography of selected papers was also performed to complete the search literature.

### 2.3 Study selection

In the first step, title/abstract selection was independently done by two reviewers (VL, LV, MD, TS,) to identify all potentially relevant articles meeting the defined eligibility criteria (Table 1). In the second step, two reviewers read the full text of each included article in the first step to determine eligibility for inclusion in this scoping review (VL, LV, MD, TS). Disagreements that arose at either stage were discussed in meetings to reach a consensus.

**Table 1:** Eligibility criteria.

	Inclusion	Exclusion
Population	Adult population (≥18 yr) with any type of cancer	Children population (<18 yr)
Concept	eHealth innovation (i.e., telemedicine, telemonitoring, teleconsultation, tele-assistance…) Technology that connects an individual to health professionals Addresses equity through access to or use of TMs.	Study unrelated to eHealth innovation Technology that connects health professional to health professional (e.g., EMR…)
Context	European countries	Countries outside the European Union
Type of studies	All original studies	All type of review, research protocol, case study, Abstract, conference
Year of publication	Published between 2018 and 2023	Published before 2018
Language	English	Other language than English
Other	Human	Animals

### 2.4 Data extraction

Study data were extracted by one reviewer (VL) according to a standardized data extraction form, then the extractions were independently checked by a second reviewer (LV) for identification and correction of inaccuracies. All discrepancies were discussed until a consensus was reached. The following data were extracted: Authors, year of publication, study information (incl. Study design, aim of the study, description of the population), eHealth innovation (e.g., telemedicine, telemonitoring, teleconsultation), cancer care pathway (e.g., prevention, screening, follow-up, survivorship…), eHealth tools (e.g., website, application medical devices…) and health equity data, i.e., all the factors over which individuals have little or no control but which can influence the incidence of disease, health outcomes and access to healthcare (19).

### 2.5 Analysis of results

The results were mapped according to the PROGRESS-Plus framework to identify population and individual characteristics across which health inequities may exist (19), as recommended by Cochrane (20). PROGRESS-Plus stands for Place of residence, Race/ethnicity/culture/language, Occupation, Gender/sex, Religion, Socioeconomic status and Social capital, and “plus” captures other personal characteristics associated with discrimination (e.g., age or disabilities) (19). The framework allows the extraction of health equity relevant data from the scientific studies identified through the literature search in the context of TM. These factors have been categorized as barriers, neutral or facilitators, where barriers are defined as factors that hinder, limit or prevent, neutral as factors that have no influence and facilitators as factors that favor, facilitate or help the access and/or the use of TMs. These factors were identified by thematic analysis. The quality of the studies included in this review and quantitative data such as effect sizes were not taken into account.

## 3 Results

### 3.1 Study selection

**Figure 1:**
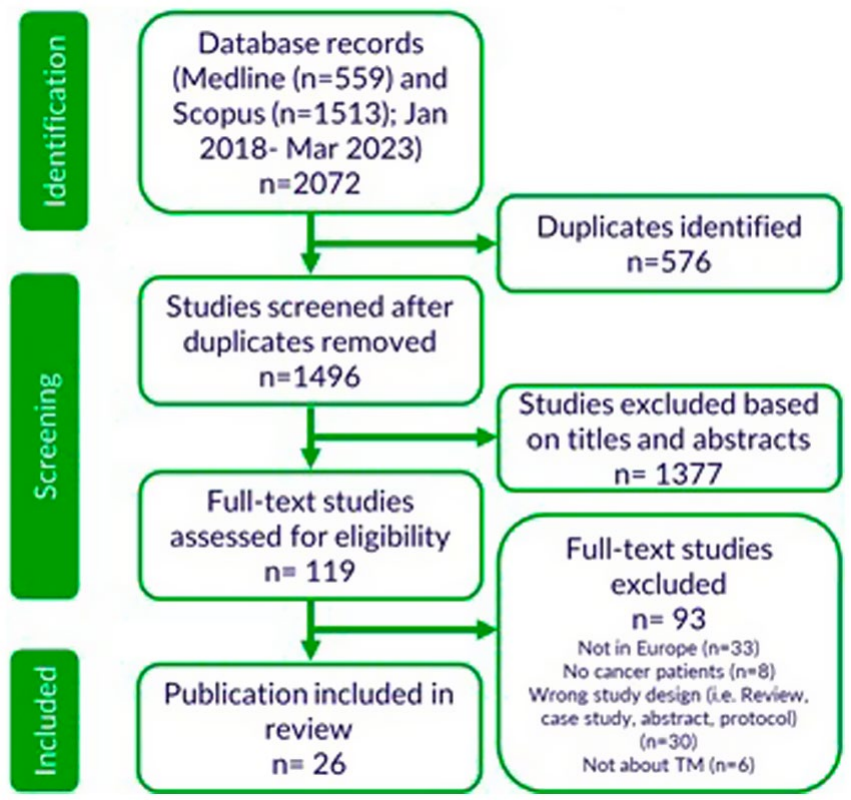
PRISMA flow chart of study selection process.

A total of 2072 references were identified through the search strategies. Following the removal of duplicate references, 1,496 were screened for eligibility based on their titles and abstracts and 119 of them were further assessed based on their full texts. Twenty-six out of 119 studies were selected for inclusion in our scoping review. A flowchart of study selection is available in Figure 1.

### 3.2 Study characteristics

All included studies were original studies (i.e., no review, no systematic reviews, no meta-analyses) and were published between 2018 and 2023. Of the 26 studies included, eight were qualitative studies (focus group discussions, interviews, thematic analyses, etc.), five used a mixed-method (i.e., quantitative and qualitative) and 13 were epidemiological studies (cross-sectional, interventional, observational, etc.). Most studies come from Western Europe: The Netherlands (21–28), Denmark (29–32), Germany (33–36), Spain (37–40), Ireland (41–43), Belgium (44) and Portugal (45). Eleven studies focused on patients with all types of cancer (21–24, 26, 29–32, 36, 41), 10 focused on breast cancer (25, 35, 37–40, 42, 44–46) and four on other cancer types (27, 28, 34, 43). Eight studies reported data about TM (22, 23, 25, 27, 39–41, 43–46), eight reported data about teleconsultation specifically (21, 26, 31, 32, 36, 38, 42) and seven reported data about access and use of telemonitoring services (24, 28, 34, 35, 37, 38). Regarding the eHealth tool, as defined in the methods section, the majority (*n* = 14) of the studies described data about intervention using mobile phones (application or phone call) (24, 25, 27, 31, 32, 35–42, 45, 46), nine using a web-based digital tool (21–23, 26, 28, 34, 42–44) and two using smartwatches (24, 42). Finally, studies reporting data on eHealth applications, across the cancer care continuum, mainly concern follow-up (*n* = 12) (22–24, 28, 31, 32, 34–36, 38, 43, 46) and survivorship (*n* = 12) (25, 26, 29, 30, 37, 39–42, 44–46) stages of the patient’s care pathway. These different studies are described in Table 2.

**Table 2:** Characteristics of the 26 included studies conducted in European countries among adult cancer patients.

Reference, Country	Study design	Aim of the study	Participants: (cancer types, sample size, gender and age)	eHealth innovation (Telemedicine, teleconsultation, telemonitoring)	Cancer care pathway (Prevention, screening, diagnosis, follow-up (treatment or symptoms management), survivorship, end-of-live or palliative care)	eHealth tools (Software, website, mobile/mHealth, application, smartwatches…)	Description
Rossen et al. (29), Denmark	Cross-sectional study	The aim is to get insight of how cancer survivors grouped by their readiness for technology are receptive toward using technology in connection with exercise to propose how services can be tailored to the groups characteristics.	All types of cancer *N* = 305 Women: 70.8% Men: 29.2% Age median: 60 yrs.	Not applicable	Survivorship, rehabilitation	Not applicable	No tool description. The study does not test a specific tool but assesses the health technology readiness of a population. Also the individuals’ receptiveness to use technology in a rehabilitation context and their readiness for health technology.
Rossen et al. (30), Denmark	Qualitative study	The aim of this qualitative study was to explore cancer survivors’ receptiveness to using digital technology as a mode of support to increase their physical activity in a municipality-based cancer rehabilitation setting.	All types of cancer *N* = 11 Women: 72.7% Men: 27.2% Age range: 32–82 yrs.	Not applicable	Survivorship, rehabilitation	Not applicable	No tool description. The study does not test a specific tool but explores the cancer survivor’s receptiveness to using digital technology as a mode of support to increase their physical activity, in a rehabilitation setting.
Stege et al. (33), Germany	Cross-sectional study	The aim of this study is to investigate the patients’ primary sources of health-related information as well as their self-proclaimed eHealth literacy.	Skin cancer *N* = 714 Women: 40.9% Men: 50.4% Unknow: 8.7% Age range: 18–89 yrs	Not applicable	All levels of eHealth application	All types of eHealth tools	No tool description. The study explores the usage of internet and other electronical devices.
Sungur et al. (21), Netherlands	Qualitative study	This study aims to systematically develop, implement, and conduct a pilot evaluation of a web-based oncological module that can be integrated into the Health Communicator to stimulate patient participation among older Turkish-Dutch and Moroccan-Dutch patients with cancer.	All types of cancer *N* = 27 Women: 67.0% Men: 33.3% Age mean: 63 yrs	Teleconsultation	All levels of eHealth application	Web-based digital tool	Health Communicator. The health communicator is a web-based digital tool that aims to resolve language barriers between non-western patients with low dutch language proficiency and their health care professionals. The tool is used to collect patient medical anamnestic data via digital questionnaires and to provide educational videos for patients in multiple languages about their illness.
Van der Hout et al. (22), Netherlands	Interventional study	The aim of this study was to investigate potential moderating factors, including socio-demographic, clinical, and personal factors, HRQOL, symptoms, and need for supportive care on the efficacy of Oncokompas on HRQOL, symptoms and patient activation.	All types of cancer *N* = 625 Women: 51.0% Men: 49.0% Age mean: 63 yrs	Telemedicine	Follow-up and survivorship	Web-based application	OncoKompas. This app supports cancer survivors to monitor their HRQL and cancer-generic and tumor specific symptoms. OncoKompas provides personalized feedback and information based on scores from PROMs, and a tailored overview of supportive care.
Yilmaz et al. (23), Netherlands	Qualitative study	The aim of this study is to gain insight into (1) the unfulfilled instrumental and affective needs of Turkish-Dutch and Moroccan-Dutch older cancer patients/survivors, (2) the barriers perceived by healthcare professionals in fulfilling these needs, and (3) how the Health Communicator, a multilingual eHealth tool, can support the fulfillment of patients’/survivors’ needs, and decrease professionals’ barriers.	All types of cancer *N* = 19 Women: 68.4% Men: 31.6% Age mean: 69 yrs.	Telemedicine	All levels of eHealth application	Web-based digital tool	Health Communicator. The health communicator is a web-based digital tool that aims to resolve language barriers between non-western patients with low Dutch language proficiency and their health care professionals. The tool is used to collect patient medical anamnestic data via digital questionnaires and to provide educational videos for patients in multiple languages about their illness.
Monteiro-Guerra et al. (37), Spain	Qualitative study	The aim of this study is to explore insights from breast cancer survivors on motivational and personalization strategies to be used in physical activity coaching apps and interventions.	Breast cancer *N* = 14 Women: 100.0% Men: 0.0% Age range: 43–69 yrs	Telemonitoring	Survivorship	Mobile application	Physical activity coaching apps
Medina et al. (38), Spain	Quasi-experimental study	The aim of this study is to examine the feasibility of ICOnnecta’t in a sample of target users during its first-year implementation. Secondary aims were to assess the psychosocial status of patients and measure their evolution in the first months within the program.	Breast cancer *N* = 189 Women = 100.0% Men = 0.0% Age mean: 54 yrs	Teleconsultation Telemonitoring	Follow-up	Mobile application	ICOnnecta’t ICOnnecta’t is an eHealth program addressed to cancer patients, to offer them a digital intervention through an app to build wellbeing and reduce psychosocial risks during the cancer journey. ICOnnecta’t consists of four care levels, provided according to users’ distress: screening and monitoring, psychoeducation campus, peer-support community, and online-group psychotherapy.
Martin et al. (46), France	Qualitative study	The aim of this study is to explore representations, levers, and barriers to physical activity and mHealth interventions among patients with breast cancer and cancer-related fatigue. Our overarching goal was to explore mHealth as a facilitator to increase physical activity in patients with fatigue after breast cancer.	Breast cancer *N* = 9 Women: 100.0% Men: 0.0% Age median: 47 yrs	Telemedicine	Follow-up and survivorship, rehabilitation	mHealth	Kiplin developed an mHealth group challenge that provides patients the opportunity of engaging in virtual exercise group challenges. It consisting of a (1) competitive virtual exercice group activity, (2) participating in a daily chat network with other partients, and (3) access to physical activity information and feedback.
Jonker et al. (24), Netherlands	Observational study	The aim of this study is to identify technological and patient-related barriers to inclusion of older patients in a clinical eHealth study.	All types of cancer N = 151 Women: 51.7% Men: 48.3% Age mean: 74 yrs	Telemonitoring	Follow-up	Mobile application Smartwatches	A mobile application connected to various electronic monitoring devices. Physical activity had been measured using an accelerometer-based wearable activity monitor (Fitbit).
Trabjerg et al. (31), Denmark	Observational study	The aim of this study is to analyze video consultations from the user’s perspective (patients and doctors), based on three surveys of patients enrolled in the intervention group, and their oncologists and GPs.	All types of cancer *N* = 44 Women: 52.0% Men: 48% Age mean: 65 yrs	Teleconsultation	Follow-up	Video call	Video call: multidisciplinary video consultation involving GP, oncologist and patients.
Kjeldsted et al. (32), Denmark	Cross-sectional study	The aim of this study is to examine patient-related and cancer-specific characteristics associated with experiences with teleconsultation among patients with cancer during the COVID-19 pandemic.	All types of cancer *N* = 792 Women: 72.0% Men: 28.0% Age median: 68 yrs	Teleconsultation	Follow-up	Telephone	No description available
Signorelli et al. (39), Spain	Mixed-methods study	The aim of this study is to examine the potential acceptability and feasibility of a mobile-based intervention to promote physical activity in patients with breast cancer; assess usability and other aspects of the user experience; and identify key considerations and aspects for future improvements, which may help increase and sustain acceptability and engagement	Breast cancer *N* = 4 Women: 100.0% Men: 0.0% Age range: 35–61 yrs	Telemedicine	Survivorship	Mobile application	Physical application coaching mobile app. The aim of the app is to increase the physical activity of the breast cancer survivors including behavioral and motivational aspects.
Melissant et al. (25), Netherlands	Exploratory study	The aim of this study is to investigate the feasibility of Oncokompas including the BC module among BC survivors by (1) investigating adoption (intention to use Oncokompas), usage (actual use of Oncokompas), and user satisfaction; (2) exploring possible socio-demographic and clinical factors, and HRQOL that may influence user satisfaction; and (3) interviewing BC survivors on possible barriers and facilitators of the feasibility of Oncokompas.	Breast cancer *N* = 68 Women: 100.0% Men: 0.0% Age mean: 56 yrs	Telemedicine	Survivorship	Mobile application	OncoKompas. This app aims to increase the knowledge of cancer survivors on the impact of cancer and its treatment on various aspects of their personal HRQOL, and to facilitate access to supportive care.
Ciria-Suarez et al. (40), Spain	Observational study	The aim of this study is to describe and assess the use of the educational section of ICOnnecta’t (virtual campus, level 2) in a sample of recently diagnosed BC patients during the first 2 years of the ecosystem’s implementation	Breast cancer *N* = 234 Women: 100.0% Men: 0.0% Age mean: 51 yrs	Telemedicine	Follow up and survivorship	Mobile application	ICOnnecta’t. ICOnnecta’t is an eHealth program addressed to cancer patients, to offer them a digital intervention through an app to build wellbeing and reduce psychosocial risks during the cancer journey. ICOnnecta’t consists of four care levels, provided according to users’ distress: screening and monitoring, psychoeducation campus, peer-support community, and online-group psychotherapy.
Haberlin et al. (41), Ireland	Mixed-methods study	The aim of this study is to explore perspectives of cancer survivors toward the concept of an eHealth-based physical activity program.	All types of cancer *N* = 102 Women: 52.9% Men: 47.1% Age mean: 65.5 yrs	Telemedicine	Survivorship	Mobile application	Physical activity program via an app.
Kiderlen et al. (34), Germany	Observational study	The aim of this study is to identify barriers met during the process and to investigate adherence and perceptions of patients as well as involved health care personnel.	Myeloma cancer *N* = 11 Women: 36% Men: 64% Age median: 65 yrs	Telemonitoring	Follow-up	Web-based / mobile application	Web-based ePRO monitoring tool on their own mobile device or computer with messenger service (patients were able to send requests and information to the clinic).
Graf et al. (35), Germany	Observational	The aim of this study is to analyze the acceptance and evaluation of a tablet-based ePRO app for breast cancer patients and to examine its suitability, effort, and difficulty in the context of HRQoL and sociodemographic factors.	Breast cancer *N* = 106 Women: 100.0% Men: 0.0% Age mean: 52 yrs	Telemonitoring	Follow-up	Web-based application	Web-based solution (PiiA: Patient interactively informs doctor) to collect data about HRQoL.
Deuning-Smit et al. (26), Netherlands	Qualitative study	The aim of this study is to identify barriers and facilitators for implementing the evidence-based blended SWORD intervention in routine psycho-oncological care.	All types of cancer *N* = 19 Women: 73.7% Men: 26.3% Age mean: 48 yrs	Teleconsultation	Survivorship	eHealth platform	SWORD - The Survivors’ Worries of Recurrent Disease. SWORD intervention is a blended psychological intervention for fear of cancer recurrence based on cognitive behavioral therapy. It compromises eight sessions with a psychologist accompanied by an interactive eHealth platform with psycho-education and at home exercises.
Brennan et al. (43), Ireland	Mixed-methods study	The aim of this study is to examine the feasibility of implementing ReStOre@Home, an online 12-week multidisciplinary rehabilitation program consisting of aerobic and resistance exercise, dietetic counseling, and education sessions, which aims to improve physical fitness, nutritional status and quality of life in UGI cancer survivors.	Stomach, esophagus and lung cancer *N* = 12 Women: 8% Men:92% Age mean: 65 yrs	Telemedicine Teleconsultation	Follow up and survivorship	Web-based eHealth platform	ReStOre@Home. ReStOre@Home is a 12-week telehealth exercise and nutrition rehabilitation program for survivors of esophago-gastric cancer. ReStOre@Home was run via a Digital Therapeutics Platform created by Salaso Health Solutions Ltd. (Kerry, Ireland). The evidence-based and clinically-tested digital therapies platform allowed us to host videocalls (one-to-one and group), and provide exercise pre-scription and appointment scheduling, which enabled a reliable and user-friendly delivery of telehealth services.
Brennan et al. (42), Ireland	Qualitative study	The aims of this study were to explore patients’ rehabilitation experiences and unmet needs during home rehabilitation after breast cancer surgery and to understand their experiences of mHealth technology and the requirements they desire from an mHealth system.	Breast cancer *N* = 10 Women: 100.0% Men: 0.0% Age range: 35–74	Teleconsultation	Survivorship	mHealth tool	Use of various mHealth tool: wearable device, smartphone, guided exercise app, mindfulness app, cancer related podcast,…
Sangers et al. (27), Netherlands	Qualitative study	The aim of this study is to explore the perceived barriers and facilitators toward mHealth apps for skin cancer screening among the Dutch general population	Skin cancer *N* = 27 Women: 68.0% Men: 32.0% Age median: 25 yrs	Telemedicine	Screening	Mobile/mHealth application	mHealth application allow users to instantly receive a risk assessment of a skin lesion by taking a smartphone camera photo.
Vogel et al. (36), Germany	Observational study	The aim of this study is to implement a web-based symptom and quality of life (QoL) assessment to address patients’ attitudes and willingness to use mHealth tools. The study also aims to evaluate sociodemographic parameters that could influence patients’ opinions.	All types of cancer *N* = 219 Women: 59.0% Men: 41.0% Age median: 33 yrs	Telemonitoring	Follow-up	Web-based applications	Web-based application to assess symptoms and QoL.
De Groef et al. (44), Belgium	Mixed-methods study	The aim of this study is to develop and test a personalized eHealth intervention containing a pain science education program and self-management strategies regarding pain and pain related functioning, tailored to the needs of breast cancer survivors	Breast cancer *N* = 29 Women: 100.0% Men: 0.0% Age mean: 51 yrs	Telemedicine	Survivorship	Web-based eHealth platform	PECAN for pain education after cancer collaborative. The aim of the eHealth intervention is to provide an understanding of the target concepts, as well as familiarity with cognitive and self-management skills to manage the pain experience and pain-related functioning. Possibility of personalisation of the program.
Mendes-Santos et al. (45), Portugal	Mixed-methods study	The aim of this study is to develop iNNOV Breast Cancer (iNNOVBC), a guided, internet-delivered, individually tailored, acceptance and commitment therapy–influenced cognitive behavioral intervention program aiming to treat mild to moderate anxiety and depression in BCSs as well as to improve fatigue, insomnia, sexual dysfunction, and health-related quality of life in this group. This study also aims to evaluate the usefulness, usability, and preliminary feasibility of iNNOVBC.	Breast cancer *N* = 11 Women: 100.0% Men: 0.0% Age median: 48 yrs	Telemedicine	Survivorship	Web-based application	iNNOV Breast Cancer. This is a guided, internet-delivered, individually tailored, ACT (Acceptance and commitment therapy) influenced CBT (Cognitive behavioral therapy) program developed to treat mild to moderate anxiety and depression in BCs, as well as, to improve fatigue, insomnia, sexual dysfunction, and HRQOL.
Qaderi et al. (28), Netherlands	Observational study	The aim was to examine patient acceptability and costs of a new remote follow-up regimen for patients with colorectal cancer.	Colorectal cancer *N* = 118 Women: 42.0% Men: 58.0% Age median: 68 yrs	Telemonitoring	Follow-up	Web-based eHealth platform	A remote follow up plan for curatively treated patients with CRC. Patients have access to their test results, are supported and empowered with self-management information, and have access to telemedicine applications such as video-consultation, text messaging, and telephone services to contact the hospital.

### 3.3 Synthesis of the results

The results presented below are based on thematic analyses from studies exploring the implementation of TMs. Our main aim was to map factors influencing access to and use of TMs, regardless of the type or quality of study, quantitative information (e.g., effect size) is not presented. According to the PROGRESS-Plus acronym, all factors, categorized as barriers, neutral and facilitators, to the access and use of TM services are shown in Table 3 and described in more detail below.

**Table 3:** Barriers and facilitators to the access to and use of telemedicine services according to the PROGRESS-PLUS framework as reported in 26 studies conducted in European countries among adult cancer patients.

PROGRESS-PLUS framework	Barriers		Neutral		Facilitators	
	Access to TMs	Use of TMs	Access to TMs	Use of TMs	Access to TMs	Use of TMs
Place of residence	No evidence from included literature	No evidence from included literature	No evidence from included literature	Distance between home and hospital (32)	Available everywhere without geographical limitations (43)	Reduction of travel time to clinic or visit to hospital (26, 28)
Race, ethnicity, culture and language	Language: limited to national languages (22, 24–31, 33–35, 37, 40, 44, 46)	Culture (23) Language (23, 24)	No evidence from included literature	No evidence from included literature	Language: tool developed to facilitate the communication with the patients (21, 23)	Language: tool developed to facilitate the communication with the patients (21, 23)
Occupation	No evidence from included literature	Retired person (32)	No evidence from included literature	Occupation (22, 25, 38, 40)	No evidence	Workers: available anytime (43)
Gender, sex	No evidence from included literature	Being a women (24)	No evidence from included literature	Being a men or women (22, 28, 33, 36)	Being a women (33)	Being a men (32)
Religion	No evidence from included literature	Religion and culture (23)	No evidence from included literature	No evidence from included literature	No evidence from included literature	No evidence from included literature
Education	Low level of digital skills (25, 27, 40)	Low level of education (23, 28, 29, 32, 33) Low digital skills or (e-)health literacy (21, 23, 25, 26, 28, 29, 32, 33, 37, 39, 41, 43, 44, 46)	No evidence from included literature	Level of education (22, 25, 36)	No evidence	Higher level of education (28, 29, 33, 35, 37, 39, 44) Good digital skills or high health and/or (e-)health literacy (22, 23, 26, 28, 35, 36, 39, 42, 43, 45)
Socioeconomic status	Low SE level: no mobile device, computer or access to internet (22, 24, 26–29, 33, 34, 36, 40, 43, 46) Additional costs (26)	Low SE level (27, 32, 33, 38)	No evidence from included literature	SE level (36)	Having own smartphone (29, 34, 37, 42)	Reimbursement or no/low cost (27) Reduction of cost (28)
Social capital	No social support (43)	Living alone (24, 29, 32) Need for social interaction (28)	No evidence from included literature	Social support (22, 25, 38, 40)	No evidence from included literature	Need for more social contact (27, 41–43, 46)
Plus: age	No evidence from included literature Internet access is age depending (33)	Older participants (21, 23, 24, 29, 33, 42)	No evidence from included literature	Age (22, 25, 28, 35, 38, 40)	No evidence from included literature	Younger participants (21, 23, 29, 33, 36) Older participants (32)
Plus: disability or complex health needs	Having comorbidities (37, 43), limited cognitive function (22, 24, 28–30, 37, 38, 40, 41)	Presence of comorbities (28, 29, 32) Smoker (29) Poly-medication (24) Frailty (24) Anxiety (32) Type of cancer: breast (32) Stage of the disease (more advanced) (28)	No evidence from included literature	Presence of comorbidities (22), stage of the disease (36, 38) or time since diagnosis (25, 36, 40)	Having received chemotherapy or radiotherapy after surgery (25)	Reduce the risk of infection/contamination (43) Type of cancer: lung, urological or gastrointestinal (32)

#### 3.3.1 Place of residence

Place of residence describes the differences in access to and use of TMs between rural, urban, and inner city places. This element is also defined by the particular region, town or community in which a person lives (19). Four studies out of 26 have investigated the role of place of residence in TM access and use.

##### 3.3.1.1 Access

While TM service are seen as a lever for reducing the health inequalities associated with geographical location facilitating access to healthcare for cancer patients. One study emphasized that one advantage of TM was that it was available everywhere without any geographical limitations (43).

##### 3.3.1.2 Use

Two studies underlined the reduction of travel time to the hospital (26, 28) as facilitators of the use of TMs. However, one study showed no effect of the distance between home and hospital on the use of TMs (32).

#### 3.3.2 Race, ethnicity, culture and language

This element refers to the racial, ethnic, cultural and language background of people which may influence their access to health care and health outcomes (19).

##### 3.3.2.1 Access

Whereas TM could facilitate communication with cancer patients regardless of their origin or language, almost all reviews reported that the language of the patient may influence access to TM service as few of them offer access regardless of the language preference (22, 24–31, 33–35, 37, 40, 44, 46).

##### 3.3.2.2 Use

Only two European studies specifically developed a tool to facilitate communication with patients, specifically migrants, regardless of the language (21, 23).

#### 3.3.3 Occupation

Occupation refers to different situations including out of work, underemployment, informal workers and unsafe working environments (19). From cancer diagnosis to cancer survivor, the patient’s care pathway can be long and affected by the occupational status of the patient.

##### 3.3.3.1 Access

No studies included in this scoping review have looked at the access to TM services by occupational status.

##### 3.3.3.2 Use

Of the six studies that looked at the influence of TM on occupation, one study showed that being retired could be a barrier to the use of TMs (32) and one study showed that being a worker may facilitate the use of TMs as it can be accessed anywhere, including the workplace (43). Four studies indicated being in employment (versus being unemployed) made no difference to TM use among cancer patients (22, 25, 38, 40).

#### 3.3.4 Gender

This element includes biological sexes and gender-based differences influencing health needs and outcomes (19). Seven studies investigated the influence of gender on TM service.

##### 3.3.4.1 Access

Only one reported that being female facilitated access to TM (33).

##### 3.3.4.2 Use

While being male appears to be a risk factor for both cancer incidence and mortality (1), gender does not seem to influence the use of TM among cancer patients, as reported in four studies (22, 28, 33, 36). One study reported being female as a barrier (24) and one being a man as a facilitator to TMs’ use (32).

#### 3.3.5 Religion

Religious affiliation can limit access to health services for a subgroup of the population because of their beliefs (19).

##### 3.3.5.1 Access

No studies included in this scoping review have explored the link between access to TM services and religious affiliation.

##### 3.3.5.2 Use

Only one out of 26 studies reported that religious beliefs and culture could be a barrier to the use of TM as illness and associated care and treatment are often seen as taboo (23).

#### 3.3.6 Education

This factor refers to the level of education of a person which influences health literacy or employment opportunities and also the health outcomes (19). A lower level of education is also a risk factor for cancer (1) and its influence on TMs was investigated in 21 studies.

##### 3.3.6.1 Access

Not having sufficient digital skills is seen as a barrier to TM services’ access (25, 27, 40).

##### 3.3.6.2 Use

Furthermore, having a higher level of education (28, 29, 33, 35, 37, 39, 44) and/or good digital skills, health and eHealth literacy (22, 23, 26, 28, 35, 36, 39, 42, 43, 45) is seen as a facilitator for TM services’ use. And vice versa, low level of education (23, 28, 29, 32, 33) and/or low digital skills or eHealth literacy (21, 23, 25, 26, 28, 29, 32, 33, 37, 39, 41, 43, 44, 46) are seen as a barrier.

#### 3.3.7 Socioeconomic status

Risk factors for cancer are more prevalent among people with lower socioeconomic levels (i.e., income and the financial resources available for an individual and the occupation) (1). Socioeconomic status is an important influence on a person’s health status and digital exclusion, which was explored in 16 studies.

##### 3.3.7.1 Access

Not having an internet connection and/or mobile devices is recognized as a barrier (22, 24, 26–29, 33, 34, 36, 40, 43, 46) and having one’s mobile device (29, 34, 37, 42) as a facilitator to accessing TMs. Furthermore, whether TM represents an additional cost may influence access to TM (26).

##### 3.3.7.2 Use

Along the same lines, having a lower socio-economic level is a barrier to the use of TMs (22, 24, 26–29, 32, 34, 36, 38, 40, 43, 46). Furthermore, whether TM reduces cost or provides reimbursement in healthcare, it will facilitate its use (27, 28).

#### 3.3.8 Social capital

This element refers to the social relationships and networks of a person, it also reflects the caregiver’s support that patients may receive or need throughout the cancer care pathway (19). Social capital was explored in 13 studies.

##### 3.3.8.1 Access

Not having sufficient social support is recognized as a barrier to TMs’ access in one study (43).

##### 3.3.8.2 Use

Among 13 studies looking at social capital, the results show that on the one hand, the lack of social contact with TM is an obstacle to their use (24, 28, 29, 32), while on the other hand, the increase in social contact through the use of TM is seen as an advantage (27, 41–43, 46). Four studies reported no influence of social capital on the use of TMs (22, 25, 38, 40).

#### 3.3.9 Plus: age

The majority of cancer patients are aged 65 and over and the prevalence of cancer is projected to rise globally with the aging population (47).

##### 3.3.9.1 Access

No studies included in this scoping review have specifically explored the influence of age on access to TM services. However, it was mentioned that having access to the internet connection is age-dependent (33).

##### 3.3.9.2 Use

Eight studies have demonstrated that age influences the use of TM service with controversial results: being older adult was shown to be a barrier in six studies (21, 23, 24, 29, 33, 42) and to be a facilitator in one study (32), being young a facilitator in five studies (21, 23, 29, 33, 36). However, no influence of age could be demonstrated in six other studies (22, 25, 28, 35, 38, 40).

#### 3.3.10 Plus: disability or complex health needs

Having one or more comorbidities and/or other health conditions may influence the prognosis for a disease such as cancer (48) and its influence was investigated in 13 studies.

##### 3.3.10.1 Access

The results of this review highlight that having comorbidities (37, 43) or limited cognitive function (22, 24, 28–30, 37, 38, 40, 41) reduce access to TM services.

##### 3.3.10.2 Use

Some studies highlighted that having comorbidities (28, 29, 32) or other health conditions (smoker (29), frailty (24), anxiety (32) or poly-medication (24)) reduce the use of TM services. The possibility offered by TMs of reducing the risk of contamination or infection is seen as a facilitator to the use of TM (43). Evidence on the potential effect of cancer type, time since diagnosis and stage of the disease on the use of TMs are not yet clear and are perceived as barriers or facilitators (25, 28, 32, 36, 38, 40).

## 4 Discussion

The eCAN JA aims to reduce inequalities in cancer care and enhance the quality of life of cancer patients by strengthening the use of digital health and TM among cancer patients in Europe (49). This JA is aligned with WHO and EU Commission initiatives to strengthen digital health, with the integration of TM services into policy framework to improve healthcare efficiency and overcome barriers in healthcare systems across Europe (50). This scoping review provides evidence on the determinants of access and use of TM service, and contributes to the development of recommendations to improve digital health strategies in Europe. More specifically, these results were integrated into the eCAN JA roadmap, a strategic document offering a comprehensive overview of current TM practices, envisioning the future of digital health in the EU, and outlining key steps to transition to a more advanced and integrated system. The eCAN roadmap proposed 16 recommendations across six intervention areas, addressing regulatory frameworks, stakeholder engagement, infrastructure development, training requirements, healthcare system integration, and outcomes evaluation. These recommendations aim to facilitate TM services adoption across Europe (49).

While the implementation of digital health in the cancer patient’s care pathway aims to reduce the burden of the disease, this can also widen existing inequities. A better understanding of these disparities will enable TM services to be used more equitably by cancer patients. On one hand, socioeconomic status, having access to the internet and a (mobile) device, and language were the most cited influential factors in accessing TM service. While, factors that influence the use of TM service include: patients’ level of education, digital skills and (e-)health literacy, social support, age and presence of comorbidities.

### 4.1 Access to telemedicine services

Access to TM refers to the ability to access the resources required for digital health (i.e., an internet connection and/or having digital devices). People with lower socioeconomic status, including difficulties with having an internet connection and not having their own mobile device, seem to have less access to TM service as well as having a language barrier or limited cognitive function. These disparities are aligned with already existing sources of health inequities among cancer patients (1, 51, 52). Having access to (mobile-) devices and the internet is a core driver of digital health equity (12). While access to the internet and/or having (mobile-) devices has improved considerably in recent years (13, 14), this remains a major problem for access to TM service (9, 12). Without good quality and affordability of internet access, patients cannot benefit from TM in all its forms (9). According to Eurostat, the statistical office of the European Union, between 80% (mainly in South-eastern Europe) to 99% (mainly in Western Europe) of European individuals have internet access (13). Only one study included in this review explored geographical and age disparities in access to TMs when it is recognized that TMs can be useful for European countries to reduce geographical disparities in cancer care (1, 53) and that internet access is age-depend (33) identifying a gap in the literature.

### 4.2 Use of telemedicine services

The use of TMs refers to the ability of different groups to access digital health technology and related resources. Beyond access, the ability to use TMs differs by the level of education with digital and health literacy, social support, age and presence of comorbidities, a finding that is consistent with the literature (51, 52); these determinants are also interconnected (51, 52, 54). Digital literacy involves both cognitive and technical skills that directly impact patients’ capacity to use TMs. In 2023, Eurostat estimated that only 55% of people in the EU aged 16 to 74 had ‘at least basic’ overall digital skills with high disparities across the EU (55). Furthermore, lower digital skills are observed among older age groups with 35% of men and 25% of women aged 65–74 having ‘at least basic’ overall digital skills (55). Older adults are the principal victims of digital exclusion. Moreover, even with good digital literacy, patients with lower health literacy and/or low education may have difficulties in seeking, using and understanding online health information which influences the use of TMs (12). Social support from family, friends or caregivers also enables the use of TMs (12).

### 4.3 Opportunities to implement more responsible and equitable telemedicine services

When used properly, TMs can help reduce inequalities among cancer patients and improve their healthcare and quality of life. However, actions are needed for equitable and responsible implementation of TMs among cancer patients but also health care providers.

First, digital health, including TMs, must be specifically designed and developed to address the specific needs of already disadvantaged groups of the population such as foreigners, migrants and people with disabilities or cognitive impairment (12). Leveraging artificial intelligence within TMs can help address inequities among cancer patients including initiatives such as: adaptive communication technologies (simultaneous translation, sign language interpretation, or real-time transcription), a visual and intuitive interface with customizable settings (font size, color, audio preference, icons, symbols…), clear and concise instructions using simple language, or tailoring the content for ethnic minority groups with culturally appropriate content (12). These developments would improve access to care for disadvantaged groups of the population by addressing the difficulties recently highlighted in *Beating Cancer Inequalities in the EU*, a report published by the Organization for Economic Co-operation and Development (OECD) like cost, language and culture barriers and poor health literacy (1). This is also in line with the actions of EBCP aiming at making the most of the digitalisation for cancer prevention by using powerful tools such as artificial intelligence and by strengthening and integrating TMs in the health and care system for cancer patients (2). Moreover, these developments will be tackled in one of the six areas of intervention: “Infrastructure and technology development” of the eCAN recommendations (49).

Second, another factor influencing the equitable use of TMs is that the digital tools developed, in both content and design, meet the needs of end-users, both patients and healthcare providers (53). Patients must be involved in the TM development process to define their expectations and establish research priorities based on their experience and knowledge. The patient’s involvement leads to a better understanding of their needs but also of their cognitive function, digital skills and (e-)health literacy for a better use and implementation of the developed tools (56). The use of TMs should provide added value for the patients and also the health care providers. The latter can intervene by accompanying patients through the process (e.g., supporting the patient’s digital skills), but also by having access to more information about the patients to adapt treatment and intervention and/or provide regular support even to remote patients. Overall, digital tools should be developed with inclusive and participatory user-centered approaches to promote health equity and improve access to cancer (53). This contributes to the achievement of the EBCP’s actions such as ensuring high standards in cancer (2) by delivering personalized care to patients, more easily accessible and supported by qualified health care providers. Finally, eCAN intervention area “Stakeholders’ engagement an awareness to prioritize the integration of telemedicine into healthcare systems” will address these developments and ensure that the end-user is at the center of the TM services development process (49).

Third, there are no major differences in the factors influencing inequity of access to and use of TMs between cancer and non-cancer patients in Europe identified in this study (11, 15). However, TMs can play a key role in addressing the many challenges associated with the complexity of the trajectory of cancer care, marked by different stages, relapses, survivorship, and end-of-life considerations (8). While some aspects of cancer treatment still require in-person visits, TMs offer greater flexibility to patients by providing access to care when it is convenient for them, reducing travel time and the distance between home and the cancer center (57). Moreover, cancer patients are also vulnerable to psychological problems with important distress, depression, anxiety or discomfort related to side effects or somatic symptoms that have a negative influence on the patient’s functioning and quality of life (58). In this way, TMs can also support the patients by providing easier and regular access to healthcare professionals through teleconsultation and telemonitoring based on the patient’s specific needs (8). Although all the articles included in this review concern cancer patients, more research is needed to better understand the specific needs and expectations of cancer patients in terms of TM compared with non-cancer patients. Finally, using the advantages of TMs to adjust treatment modalities to the specific needs of cancer patients will support EBCP’s mission (2) aiming at improving the quality of life for cancer patients survivors and carers. The eCAN areas of intervention, “Regulatory, governance and policy framework” and “Implementation & integration into healthcare systems” will seek to integrate TM services as effectively as possible into the patient’s cancer care pathway (49).

Fourth, access to digital health is more and more recognized as a determinant of health and is specifically defined as a digital determinant of health, including access to technological tools, digital literacy, and community infrastructure like broadband internet (9). These digital determinants of health interact with social determinants of health, either reducing or improving patients’ health outcomes. One of the key challenges of EBCP, established by the EU Commission, is to tackle inequalities in cancer prevention and care. In this context, the EU Cancer Inequalities Registry was set up and aimed to identify trends, disparities and inequalities in cancer prevention and care for each EU Member state. However, for the time being, no information on access to digital health is available in the monitoring tool (59). There is a need for monitoring and reporting these digital determinants of health, leading to widening inequities to help develop a good practice approach when generating policy-relevant evidence (53). This can support policymakers and help guide investment and interventions at regional, national and EU levels under EBCP (2). The eCAN area of intervention, “Evaluation and continuous monitoring” will ensure the establishment of a more transparent monitoring of access and use of TM services (49).

### 4.4 Strengths and limitations

This comprehensive scoping review on inequities in TMs among cancer patients in Europe enables to capture their specific needs in an increasingly digital world. These results also provide decision-makers with evidence to support their decisions in improving cancer care using TMs in Europe while cancer incidence is on the rise across Europe. We used PRISMA-ScR statement and the PROGRESS-plus framework to ensure the quality of our study and reporting. However, there are also some limitations, only two different bibliographic databases were consulted. Even if a manual search within the bibliography of selected papers was also performed to complete the search literature, some studies providing information on inequities to TMs may have been missed. Second, data extraction was performed by only one author. While the data was checked very carefully by a second reviewer, there is a limited risk of bias in data collection. The evidence identified comes from qualitative studies or quantitative scientific studies following rigorous methodologies and strict inclusion criteria; these variety of study designs may bring heterogeneity in the quality of studies and may limit the generalizability of our results; and underline the need for pragmatic trials with real-world data to better capture the reality on the ground. Then, some PROGRESS-Plus domains are underrepresented in this review, such as religion, culture, race, and ethnicity. This likely reflects broader gaps in the European digital health research landscape, where these equity dimensions are less frequently examined or reported. There is a need to ensure that all relevant equity dimensions are considered in future studies. Next, while the included studies highlighted key individual factors contributing to inequities in the access and use of TM services, few provided a detailed analysis of the underlying contextual or political context. This points to an important gap in the current evidence base and highlights the need for future research that examines how the national, structural and political context may shape digital equity. Finally, while the geographical distribution of studies included in this scoping review reflects the available literature, the outcomes of this study are mainly represented by Western European countries. This may limit the generalizability of findings across the broader European context and underrepresent the unique challenges faced by patients and health systems in Eastern and some Southern European countries, where digital infrastructure, funding for TM, and digital literacy programs may be less developed.

## 5 Conclusion

The findings of this scoping review highlighted the main factors influencing access to and use of TMs among cancer patients in Europe. These results will enable the development and tailoring of TMs that align more effectively with the needs and expectations of cancer patients. Better integration of patient needs in TM is necessary to enhance equity and allow a better implementation of TMs in European health and care systems aligned with different initiatives such as the European Beating Cancer Plan. Ensuring that all individuals have access to high-quality care and support regardless of their abilities, socio-economic status or age by offering more inclusive TMs. TMs should be developed and used to tackle inequities already present among cancer patients in Europe instead of widening the gap.

## Data Availability

The original contributions presented in the study are included in the article/Supplementary material, further inquiries can be directed to the corresponding author.

## Footnotes

^1^ https://osf.io/sx2yu/
