# Supplementary material for: Inequities to the access to and use of telemedicine among cancer patients in Europe: a scoping review
Source: Front Public Health. 2025 May 22;13:1483706. doi: 10.3389/fpubh.2025.1483706 (PMC12137269; doi:10.3389/fpubh.2025.1483706)
Supplement: Supplementary file 2 [file Supplementary_file_2.docx]

**Appendix 2. Search strategies**

Studies were identified via a search literature in Ovid Medline and Scopus using the selected keywords in March 2023. A combination of terms of Medical Subject Heading (MeSH) and keywords was used in the search strategy. Keywords for eHealth infrastructure, cancer and health inequalities were combined.

The filter on European countries has been applied in the Scopus database. However, the country filter was not applied in the Ovid Medline database, the selection on the basis of countries will be done during the screening of the abstracts.

1. Scopus

Date of search : 21 March 2023

1513 observations

((((TITLE-ABS-KEY (tele W/1 communication*)) OR (TITLE-ABS-KEY (rehabilitation* W/1 (tele OR remote OR e or electronic or digital* or virtual* or video or online or app*))) OR (TITLE-ABS-KEY (medic* W/1 (tele or remote or e or electronic or digital* or virtual* or video or online or app*))) OR (TITLE-ABS-KEY (monitoring W/1 (tele or remote or e or electronic or digital* or virtual* or video or online or app*))) OR (TITLE-ABS-KEY (consultation W/1 (tele or remote or e or electronic or digital* or virtual* or video or online or app*))) OR (TITLE-ABS-KEY ("telepathology")) OR (TITLE-ABS-KEY ("telemedicine")) OR (TITLE-ABS-KEY ("telerehabilitation")) OR (TITLE-ABS-KEY ("telehealth")) OR (TITLE-ABS-KEY ("teleradiology")) OR (TITLE-ABS-KEY ("telecare")) OR (TITLE-ABS-KEY ("teleconference")) OR (TITLE-ABS-KEY ((health or medic*) W/1 (mobile or tele or app* or digital* or e or remote or virtual*))) OR (TITLE-ABS-KEY ("mhealth")) OR (TITLE-ABS-KEY(digital W/1 healthcare)) OR (TITLE-ABS-KEY ((electronic) W/1 (health or medic*) W/1 (record* or tool*))) OR (TITLE-ABS-KEY (eprom* or (electronic w/1 patient W/1 reported w/1 outcome)))))) AND ((TITLE-ABS-KEY(neoplas* OR malign* OR tumo* OR cancer* OR adenom* OR fibrom*))) AND (((TITLE-ABS-KEY(((rural W/1 health) and (equity or inequit* or equality or inequalit* or disparit*))) OR TITLE-ABS-KEY(((health or healthcare or "health care") W/2 (equity or inequit* or equality or inequalit* or disparit*))) OR TITLE-ABS-KEY(((Underserved W/1 area* W/1 medically) or (Underserved W/1 population* W/1 medically))) OR TITLE-ABS-KEY((underserved W/1 patient*)))) or (((TITLE-ABS-KEY(((Socioeconomic or socio-economic or sociological or economic) W/1 (factor* or status or determinant*))) OR TITLE-ABS-KEY((Social W/1 (inequalit* or isolation or exclusion or vulnerability))) OR TITLE-ABS-KEY((Population* W/1 (high W/1 income))) OR TITLE-ABS-KEY((Population* W/1 (low W/1 income))) OR TITLE-ABS-KEY((Economic W/1 (factor* or status))) OR TITLE-ABS-KEY((Class* W/1 (population* or social))) OR TITLE-ABS-KEY(poverty) OR TITLE-ABS-KEY(((social or sociological) W/1 (trait* or attribute* or characteristic* or factor* or phenomena or context* or ecology* or environment* or condition*))) OR TITLE-ABS-KEY(((Minorit* W/1 (ethnic or racial or group*)) and (equity or inequit* or equality or inequalit* or disparit*))) OR TITLE-ABS-KEY((determinant* W/1 (Social or structural))) OR TITLE-ABS-KEY((Factor* W/1 sociodemographic)) OR TITLE-ABS-KEY((Gender W/1 (equity or inequit* or equality or inequalit* or disparit*))) OR TITLE-ABS-KEY((Social W/1 deprivation*)) OR TITLE-ABS-KEY((Social W/1 deprivation* W/1 (index or indices))) OR TITLE-ABS-KEY((Population W/1 (vulnerable or underserved or rural or sensitive or disadvantaged))))) AND (TITLE-ABS-KEY((health or healthcare or "health care")))) or ((TITLE-ABS-KEY((health W/1 (education or literacy))) OR TITLE-ABS-KEY((health W/1 (education or literacy)))))) AND ( LIMIT-TO ( PUBYEAR,2023) OR LIMIT-TO ( PUBYEAR,2022) OR LIMIT-TO ( PUBYEAR,2021) OR LIMIT-TO ( PUBYEAR,2020) OR LIMIT-TO ( PUBYEAR,2019) OR LIMIT-TO ( PUBYEAR,2018) ) AND ( LIMIT-TO ( LANGUAGE,"English" ) )

Database: Ovid MEDLINE(R) <1946 to March 17, 2023>

Search Strategy:

--------------------------------------------------------------------------------

1 *Telemedicine/ (31212)

2 *Remote Consultation/ (4324)

3 *Telepathology/ (774)

4 *Telerehabilitation/ (886)

5 (tele adj1 communication).ti,ab,kf. (12)

6 (rehabilitation* adj1 (tele or remote or electronic or digital* or virtual* or video or online)).ti,ab,kf. (522)

7 (medic* adj1 (tele or remote or electronic or digital* or virtual* or video or online or app*)).ti,ab,kf. (49784)

8 (monitoring adj1 (tele or remoteor electronic or digital* or virtual* or video or online)).ti,ab,kf. (2077)

9 (consultation adj1 (tele or remote or electronic or digital* or virtual* or video or online)).ti,ab,kf. (1322)

10 (telepathology or telemedicine or telerehabilitation or telehealth or teleradiology or telecare or teleconference).ti,ab,kf. (27827)

11 (mhealth or ehealth or econsultation or emonitoring or emedicine or erehabilitation).ti,ab,kf. (9637)

12 (digital adj1 healthcare).ti,ab,kf. (243)

13 ((electronic or computerized) adj1 (health or medical) adj1 (record* or tool*)).ti,ab,kf. (42462)

14 eprom*.ti,ab,kf. (86)

15 ((health or medic*) adj1 (mobile or tele or electronic or app* or digital* or e or remote or virtual*)).ti,ab,kf. (95068)

16 1 or 2 or 3 or 4 or 5 or 6 or 7 or 8 or 9 or 10 or 11 or 12 or 13 or 14 or 15 (139660)

17 (neoplas* or malign* or tumo* or oncolo* or cancer* or carcino* or adenom* or fibrom*).ti,ab,kf. (3574233)

18 exp Neoplasms/ (3806320)

19 exp Medical Oncology/ (29288)

20 17 or 18 or 19 (4573888)

21 exp Healthcare Disparities/ (21858)

22 Health Inequities/ (306)

23 Health Equity/ (3366)

24 Rural Health/ (23892)

25 (rural adj1 health).ti,ab,kf. (7665)

26 (equity or inequit* or equality or inequalit* or disparit*).ti,ab,kf. (148157)

27 (24 or 25) and 26 (1407)

28 (health or healthcare or "health care").ti,ab,kf. (2271482)

29 ((health or healthcare or "health care") adj2 (equity or inequit* or equality or inequalit* or disparit*)).ti,ab,kf. (41589)

30 Medically Underserved Area/ (7460)

31 (Underserved adj1 area* adj1 medically).ti,ab,kf. (435)

32 (Underserved adj1 population* adj1 medically).ti,ab,kf. (303)

33 (underserved adj1 patient*).ti,ab,kf. (587)

34 30 or 31 or 32 or 33 (8271)

35 21 or 22 or 23 or 27 or 29 or 34 (66009)

36 exp Socioeconomic Factors/ or sociological factors/ or minority groups/ or exp social conditions/ or exp social environment/ or social isolation/ or social vulnerability/ or sociodemographic factors/ or economic factors/ or economic status/ or poverty/ or social class/ or social factors/ or "social determinants of health"/ or gender equity/ or social deprivation/ (640019)

37 ((Socioeconomic or socio-economic) adj1 (factor* or status or determinant*)).ti,ab,kf. (75083)

38 (Social adj1 (inequalit* or isolation or exclusion)).ti,ab,kf. (16093)

39 (Population* adj1 (high adj1 income)).ti,ab,kf. (7)

40 (Population* adj1 (low adj1 income)).ti,ab,kf. (28)

41 (Economic adj1 (factor* or status)).ti,ab,kf. (39414)

42 (Class* adj1 (population* or social)).ti,ab,kf. (11680)

43 ((social or sociological) adj1 (trait* or attribute* or characteristic* or factor* or phenomena or context* or ecology* or environment*)).ti,ab,kf. (35475)

44 "Ethnic and Racial Minorities"/ (477)

45 (Minorit* adj1 (ethnic or racial or group*)).ti,ab,kf. (19586)

46 44 or 45 (19627)

47 46 and 26 (5527)

48 (determinant* adj1 (Social or structural)).ti,ab,kf. (16783)

49 (Factor* adj1 sociodemographic).ti,ab,kf. (9071)

50 (Gender adj1 (equity or inequit* or equality or inequalit* or disparit*)).ti,ab,kf. (6567)

51 (Social adj1 deprivation*).ti,ab,kf. (2020)

52 (Social adj1 deprivation* adj1 (index or indices)).ti,ab,kf. (151)

53 (Population adj1 (vulnerable or underserved or rural or sensitive or disadvantaged)).ti,ab,kf. (17491)

54 36 or 37 or 38 or 39 or 40 or 41 or 42 or 43 or 47 or 48 or 49 or 50 or 51 or 52 or 53 (739028)

55 54 and 28 (289997)

56 Health Literacy/ (8920)

57 Health Education/ (63450)

58 Digital Divide/ (170)

59 (digital adj1 (exclusion or divide or inclusion)).ti,ab,kf. (863)

60 (health adj1 (education or literacy)).ti,ab,kf. (47651)

61 56 or 57 or 58 or 59 or 60 (99947)

62 35 or 55 or 61 (414200)

63 16 and 20 and 62 (970)

64 limit 63 to (english language and humans and yr="2018 -Current") (559)

***************************
